# Population pharmacokinetic modeling of multiple-dose intravenous fosfomycin in critically ill patients during continuous venovenous hemodialysis

**DOI:** 10.1038/s41598-023-45084-5

**Published:** 2023-10-24

**Authors:** Tobias Hüppe, Katharina M. Götz, Andreas Meiser, Andrea de Faria Fernandes, Felix Maurer, Heinrich V. Groesdonk, Thomas Volk, Thorsten Lehr, Sascha Kreuer

**Affiliations:** 1 Department of Anesthesiology, Intensive Care and Pain Therapy, Saarland University Medical Center, Kirrberger Straße 100, 66421 Homburg (Saar), Germany; 2 Clinical Pharmacy, Saarland University, Saarbrücken, Germany; 3 Saarmetrics GmbH, Saarbrücken, Germany; 4 Department of Interdisciplinary Critical Care Medicine and Intermediate Care, Helios Clinic Erfurt, Erfurt, Germany

**Keywords:** Medical research, Infectious diseases

## Abstract

The aim of this study was to investigate the pharmacokinetics of multiple-dose intravenous (i.v.) fosfomycin in critically ill patients during continuous venovenous hemodialysis (CVVHD). Non-compartmental analysis and population pharmacokinetic modeling were used to simulate different dosing regimens. We evaluated 15 critically ill patients with renal insufficiency and CVVHD undergoing anti-infective treatment with fosfomycin in our ICU. Five grams of fosfomycin were administered for 120 min every 6 h. Plasma concentrations were determined with and without CVVHD. Pharmacokinetic analysis and simulations were performed using non-linear mixed effects modelling (NONMEM). A two-compartment model with renal and dialysis clearance was most accurate in describing the pharmacokinetics of i.v. fosfomycin during CVVHD. Population parameter estimates were 18.20 L and 20.80 L for the central and peripheral compartment volumes, and 0.26 L/h and 5.08 L/h for renal and intercompartmental clearance, respectively. Urinary creatinine clearance (CL_CR_) represented a considerable component of renal clearance. Central compartment volume increased over time after the first dose. For patients with CL_CR_ > 50 (90) mL/min and CVVHD, dosage should be increased to ≥ 15 (16) grams of i.v. fosfomycin across three (four) daily doses. Individual CL_CR_ must be considered when dosing i.v. fosfomycin in critically ill patients during CVVHD.

## Introduction

Fosfomycin is a bactericidal broad-spectrum antibiotic with a high level of tissue penetration, which is licensed for patients with severe infections^1^. It is a small and hydrophilic molecule with negligible plasma protein binding that does not undergo metabolization. Elimination occurs almost exclusively via renal clearance with glomerular filtration, which makes fosfomycin highly dialyzable^2–4^.

Maintaining defined plasma concentrations is a prerequisite for effective antimicrobial therapy and for minimizing side effects. However, critical illness may change the pharmacokinetics (PKs) of antibiotics through shifts in volumes of distribution, hypoproteinemia and reduced renal clearance. Moreover, organ replacement therapies can result in unpredictable and ineffective plasma concentrations or toxicity^5,6^. Therapeutic drug monitoring (TDM) can be used to ensure adequate exposure to anti-infective treatment in critically ill patients. However, it is relatively expensive, not commonly available, and may require long analysis times^7^. Apart from the aminoglycosides and vancomycin, it plays little role in clinical practice.

Population pharmacokinetic modeling approaches can be used to predict plasma concentrations and guide dosing regimens in individual patients. Currently available pharmacokinetic models of intravenous (i.v.) fosfomycin are based on data from non-critically ill subjects^8^ or patients with preserved renal function^9,10^. A population pharmacokinetic model for multiple-dose i.v. fosfomycin in critically ill patients with renal insufficiency undergoing renal replacement therapy has not been published yet, and clinical data to develop such a model are sparse.

We therefore conducted an observational study of multiple-dose regimes of i.v. fosfomycin in critically ill patients during continuous venovenous hemodialysis (CVVHD) and following interruption of CVVHD. Concentration–time profiles were analyzed using a non-compartmental analysis approach (NCA). In addition, a population pharmacokinetic model was developed and covariates influencing the PKs of i.v. fosfomycin were investigated. Different dosing regimens within the range of approved daily doses with and without CVVHD were simulated to detect possible over- or underdosing of i.v. fosfomycin in critically ill patients.

## Results

### Patient characteristics

In total, 15 patients (13 male, 87%) were enrolled with a mean age of 60 ± 8 years, weight of 88.5 ± 20.5 kg, and height of 176 ± 10 cm; 6 patients were anuric. Mean serum creatinine was 1.7 ± 0.9 mg/dL, 12-h urine-output was 250 ± 430 mL and urinary creatinine-clearance was 20.7 ± 44.9 mL/min. Dialysate flow rate (DFR) was 2.3 ± 0.5 L/h, blood flow rate (BFR) was 110 ± 25 mL/min, and ultrafiltration was 88 ± 83 mL/h (Table 1).

**Table 1:** Demographic Data and Clinical Characteristics.

Patient	1	2	3	4	5	6	7	8	9	10	11	12	13	14	15
Age	57	51	59	56	59	80	62	62	68	70	49	53	56	54	56
Sex	M	M	M	M	F	M	M	M	M	W	M	M	M	M	M
Weight (kg)	100	110	79	120	58	90	75	80	100	55	115	75	122	79	89
Height (cm)	181	178	176	180	160	170	175	175	173	150	187	185	180	192	172
SAPS II (Points/In-Hospital-Mortality [%])	38/21,3	49/43,8	65/76,9	52/50,7	43/30,6	52/50,7	51/48,8	79/83,8	41/26,6	33/14	48/41,5	49/43,8	79/91,9	53/53	47/39,2
Vasoactive Drugs	no	yes	yes	no	no	no	no	no	no	yes	no	no	no	no	yes
Mechanical Ventilation	no	yes	yes	no	yes	no	no	yes	no	yes	no	no	yes	no	no
CVVHD (Bloodflow [ml/min]/Dialysate Flow) [L/h])	100/2,5	100/2,5	100/2	100/2,5	100/2	100/2,5	200/4	100/2	100/2	150/3	100/2,5	100/2	100/2	100/2	100/2
ICU Survival	yes	no	yes	no	yes	yes	yes	no	yes	yes	yes	yes	yes	no	yes
Type of Infection	Urinary tract infection	Fournier's gangrene with bacteremia	Septicemia with unclear focus	Perianal fistula and abscess with septicemia	Peritonitis after abdominal surgery on the colon	Septicemia after prosthetic aortic replacement	Septicemia with unclear focus	Septic embolism after aortic replacement	Pneumonia and septic knee joint effusion	Abdominal infection with peritonitis	Abdominal infection	Sepsis with unclear etiology	Sepsis with unclear etiology	Urinary tract infection	Sepsis with unclear etiology
Pathogen	–	Streptococcus anginosus	–	–	–	–	–	Staphylococcus epidermidis	Staphylococcus aureus in knee effusion	–	Staphylococcus lugdunensis	–	Staphylococcos epidermidis	Staphylococcus capitis	–
Concomitant Antimicrobial Therapy	–	Meropenem and Clindamycin	Piperacillin/ Tazobactam	Tigecyclin	Meropenem	Meropenem	Meropenem	Vancomycin and Cotrimoxazol	Meropenem	Linezolid and Piperacillin/ Tazobactam	Piperacillin/ Tazobactam	–	–	Piperacillin/ Tazobactam	Piperacillin/ Tazobactam
Main Indication for CVVHD	Acute on chronic renal failure	Acute renal failure due to septicemia	Acute renal failure due to septicemia	Acute renal failure, Hepatorenal syndrome	Chronic renal failure with permanent dialysis	Acute renal failure	Chronic renal failure with permanent dialysis	Chronic renal failure with permanent dialysis	Acute on chronic renal failure	Acute renal failure due to septicemia	Acute on chronic renal failure	Acute on chronic renal failure	Acute renal failure due to septicaemia	Hepatorenal Syndrom	Acute renal failure after liver transplantation
Creatinine Serum (mg/dL)	1.7 ± 0.9														
Creatinine Urine (mg/dL)	22 ± 45														
Albumin (g/L)	24 ± 3.5														
Protein (g/L)	54 ± 7.6														
Urea (mg/dL)	44 ± 27														
Potassium (mmol/L)	4.3 ± 0.4														
Sodium (mmol/L)	144 ± 3.8														
Urine output in 12 h (mL)	250 ± 430														
Urine output in 24 h (mL)	480 ± 740														
CR_CL_ from 12 h urine output (mL/min)	20.7 ± 44.9														

Demographic data of 15 individual patients and clinical characteristics. SAPS = Simplified Acute Physiology Score, CVVHD = Continuous Venovenous Hemodialysis. CR_CL_ = Creatinine Clearance. Clinical characteristics are represented as mean ± SD.

**Figure 1:**
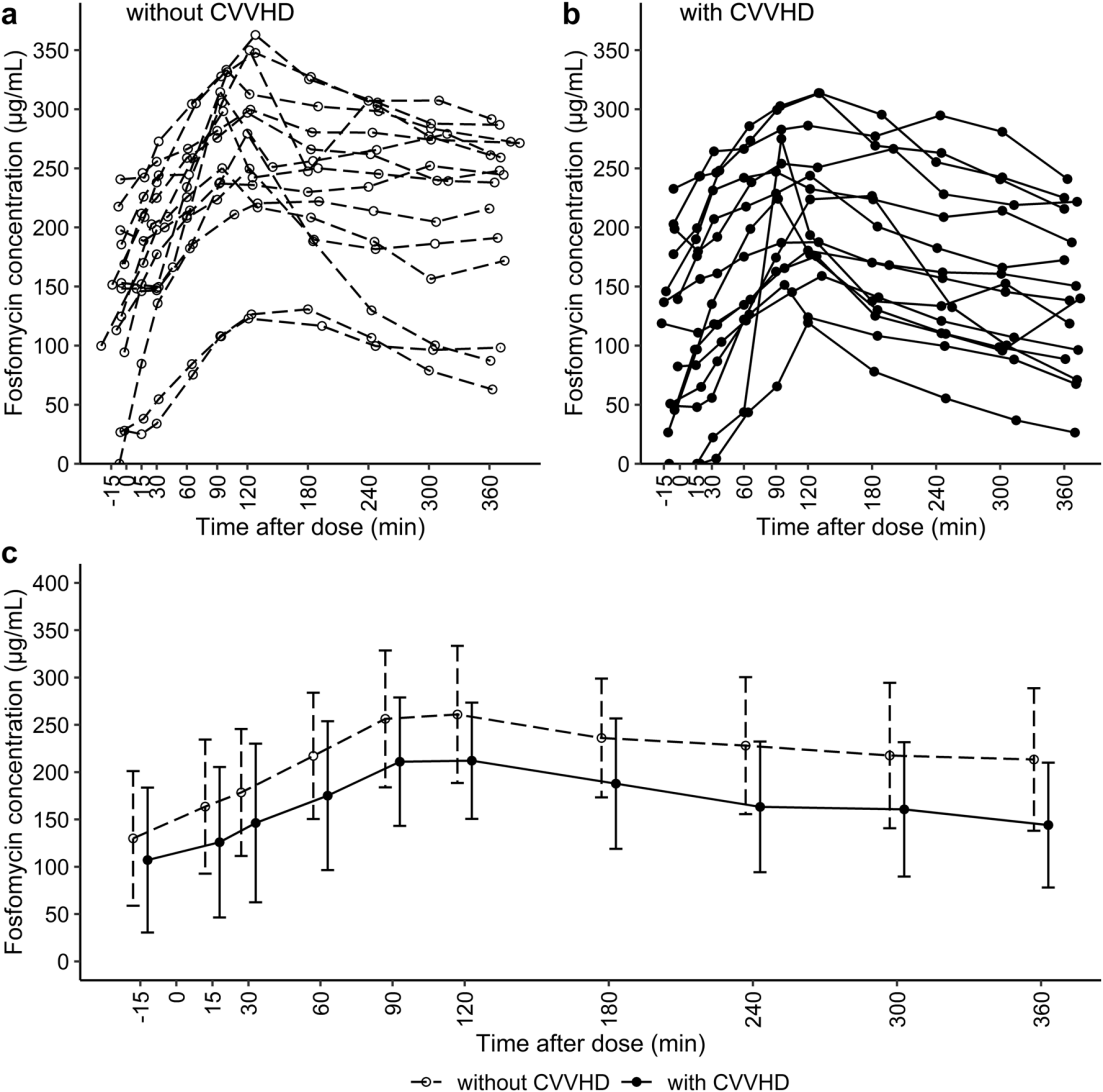
Fosfomycin plasma concentration–time profiles Fosfomycin PK study data after treatment with 5 g of i.v. fosfomycin. Individual (*n* = 15) plasma concentration–time profiles without (**a**) and with (**b**) CVVHD. **c** Mean (10th to 90th percentiles) fosfomycin plasma concentrations over a period of 360 min differentiated by CVVHD. Circles (dashed lines) and dots (solid lines) represent fosfomycin concentrations without and with CVVHD, respectively. CVVHD = continuous venovenous hemodialysis.

For the majority of patients (60%), the first series of PK measurements was obtained during CVVHD treatment, while the second series was obtained without renal replacement therapy. On average, 41.5 ± 40.3 h elapsed between the two series of PK measurements. A total of 300 concentrations of i.v. fosfomycin were determined in patient plasma and included in statistical analyses. Individual concentration–time profiles without and with CVVHD treatment are presented in Fig. 1a,b, respectively. Fosfomycin concentrations were lower during CVVHD treatment and between-subject variability was high both with and without CVVHD treatment (Fig. 1c).

### Non-compartmental analysis

**Figure 2:**
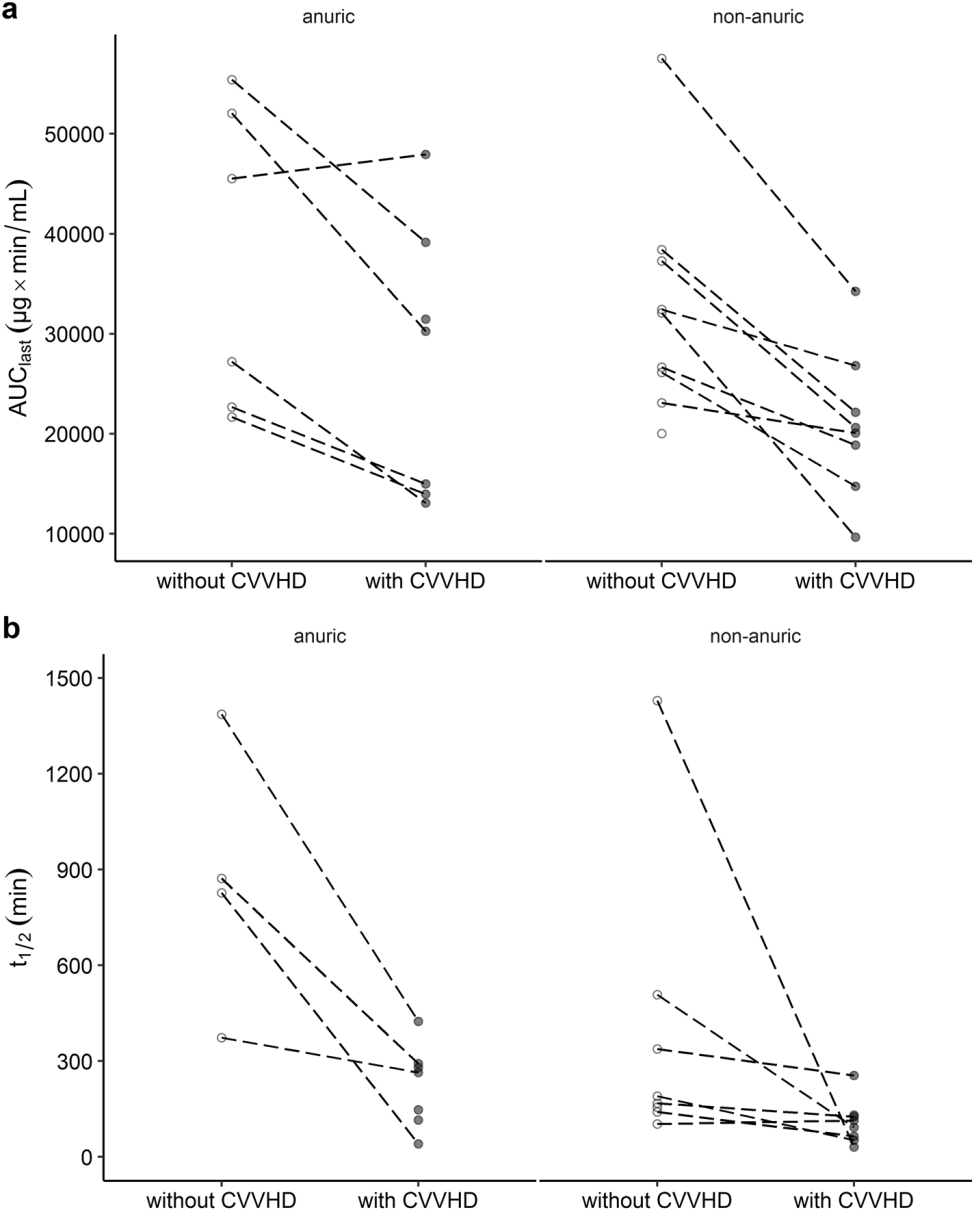
Non-compartmental analysis Non-compartmental analysis of fosfomycin plasma concentrations after treatment with 5 g of i.v. fosfomycin. Individually calculated AUC_last_ (**a)**, *n* = 15) and elimination half-life t_1/2_ (**b,** *n* = 12) in anuric and non-anuric patients. Dashed lines show paired data from one individual. Circles and dots represent NCA parameters without and with CVVHD, respectively. AUC_last_ = area under the concentration–time curve, CVVHD = continuous venovenous hemodialysis, NCA = non-compartmental analysis, t_1/2_ = elimination half-life.

AUC values were 28.7% lower under CVVHD treatment (23,869 ± 10,888 µg × min/mL, *n* = 15) compared to no CVVHD treatment (34,549 ± 12,705 µg × min/mL, *n* = 15; *p* < 0.05). Dialysis decreased elimination half-life by 51.0%, which was 541 ± 479 min without CVVHD (*n* = 12) and 162 ± 115 min with CVVHD (*n* = 15; *p* < 0.05). Figure 2a,b illustrate the effects of renal replacement therapy on AUC and t_1/2_ values using paired data of individual anuric and non-anuric patients. Similarly, c_max_ values were 17.3% lower on CVVHD (118 ± 60 µg/mL, *n* = 15; *p* < 0.05) than following discontinuation of CVVHD (144 ± 56 µg/mL, *n* = 15).

### Population pharmacokinetic modeling

**Figure 3:**
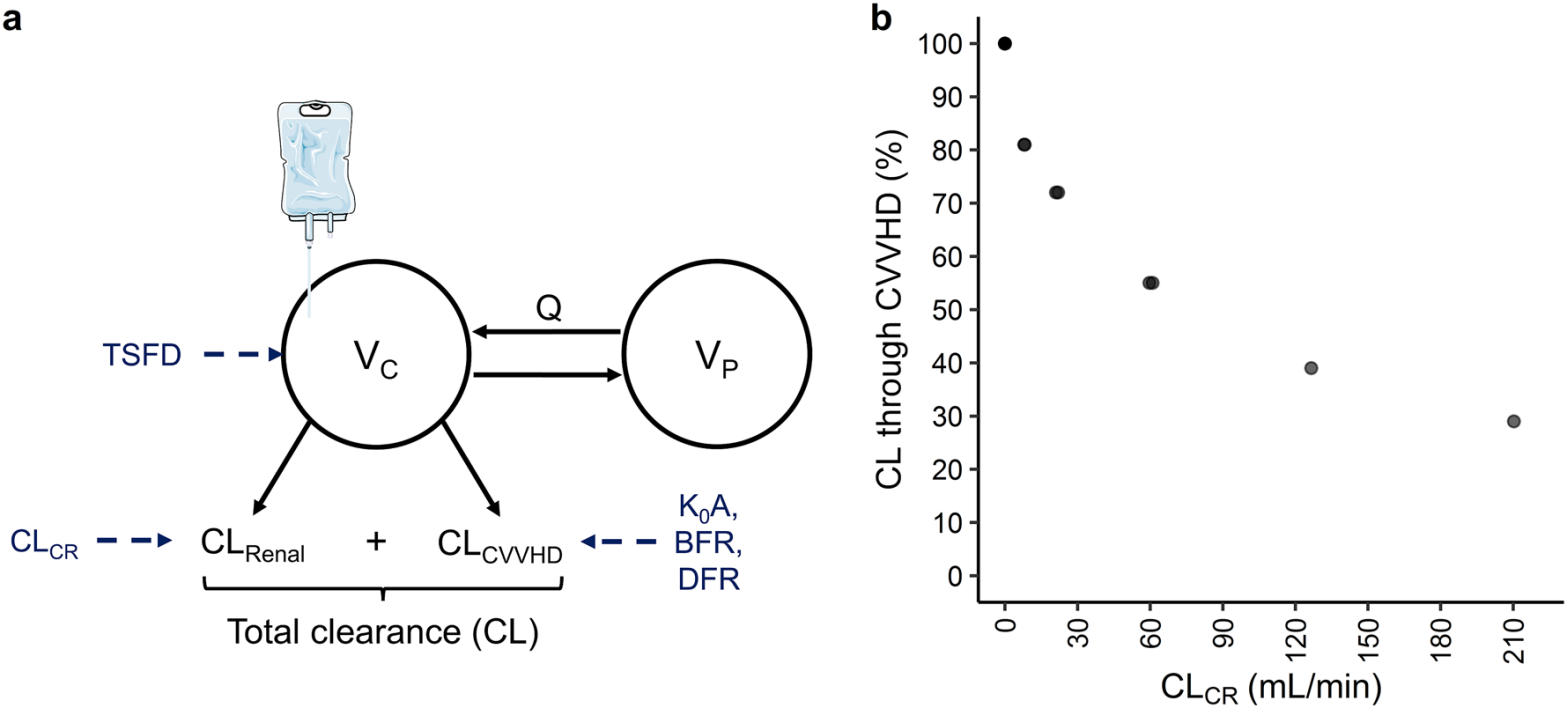
Final population pharmacokinetic model (**a**) Final population pharmacokinetic model for i.v. fosfomycin with CVVHD. Total fosfomycin clearance (CL) is the sum of renal (CL_Renal_) and dialysis clearance (CL_CVVHD_). CL_Renal_ increases with higher urinary creatinine clearance (CL_CR_). CL_CVVHD_ is dependent on the hemodialyzer mass transfer-area coefficient (K_0_A), the blood flow rate (BFR) as well as the dialysis flow rate (DFR). The central volume compartment (V_C_) increases with time since first dose (TSFD). V_P_ = peripheral compartment volume, Q = intercompartmental clearance. (**b**) Percentage of total fosfomycin clearance (CL) through CVVHD as a function of individual CL_CR_: the higher the creatinine clearance, the lower the proportion of dialysis clearance. CVVHD = continuous venovenous hemodialysis.

The PKs of i.v. fosfomycin were best described by a two-compartment model. Two concurrent clearance processes were implemented to describe i.v. fosfomycin elimination (Fig. 3a). Renal clearance (CL_Renal_) was estimated at 0.26 L/h in patients with residual diuresis. Dialysis clearance (CL_CVVHD_) was parameterized using the Michaels equation (Eq. 1)^11^:

1 $$ {\text{CL}}_{{\text{CVVHD }}} { = }\frac{{{\text{BFR}}\left( {{\text{e }}^{{\frac{{{\text{K}}_{{0}} {\text{A}}}}{{{\text{BFR}}}}{ }\left( {{\text{1 {-} }}\frac{{{\text{BFR}}}}{{{\text{DFR}}}}} \right)}} { - 1}} \right)}}{{{\text{e }}^{{\frac{{{\text{K}}_{{0}} {\text{A }}}}{{\text{BFR }}}{ }\left( {{\text{1 {-} }}\frac{{{\text{BFR}}}}{{{\text{DFR}}}}} \right){ }}} {\text{{-} }}\frac{{{\text{BFR}}}}{{{\text{DFR}}}}{ }}}{ } $$

K_0_A is the mass transfer-area coefficient of the dialysis filter used. Individual BFR and DFR values were input variables for Eq. 1 and K_0_A was estimated at 0.0288 mL/min. Dialysis clearance was fixed to zero in periods without CVVHD treatment. Total i.v. fosfomycin clearance (CL) was derived from the sum of patients’ renal and dialysis clearance. Population parameter estimates were 18.20 L and 20.80 L for the central and peripheral compartment volume, respectively, and 5.08 L/h for intercompartmental clearance. The final PK model incorporated interindividual variability in central compartment volume (V_C_). An additive error model was used to explain residual variability.

During model development, inter-individual variability (IIV) was incorporated on CL_Renal_ and V_C_. Subsequently, covariate analysis identified urinary creatinine clearance (CL_CR_) and time since first dose as significant factors influencing CL_Renal_ and V_C_, respectively. The inclusion of CL_CR_ on CL_Renal_ completely characterized the IIV on CL_Renal_. Thereafter, the inclusion of IIV on CL_Renal_ provided no longer a benefit of statistical significance (*p* > 0.05) and thus it was not included in the final model. To examine the effects of renal function and dialysis clearance on total fosfomycin clearance, the proportion of dialysis clearance within total fosfomycin clearance was calculated and correlated with urinary creatinine clearance. Figure 3b shows the decreasing percentage of dialysis clearance in total fosfomycin clearance with increasing urinary creatinine clearance. Variations in weight, creatinine clearance and serum levels of creatinine, albumin, total protein, urea, potassium, and sodium failed to adequately account for changes in central compartment volume over time. Concentration–time profiles across consecutive days of treatment with i.v. fosfomycin (days after the first dose) showed reduced i.v. fosfomycin absorption and elimination. Data were best described by increases in central compartment volume over time since first dose (TSFD). Thus, a linear correlation most accurately characterizes the continuous increase in the distribution of i.v. fosfomycin over consecutive doses.

**Figure 4:**
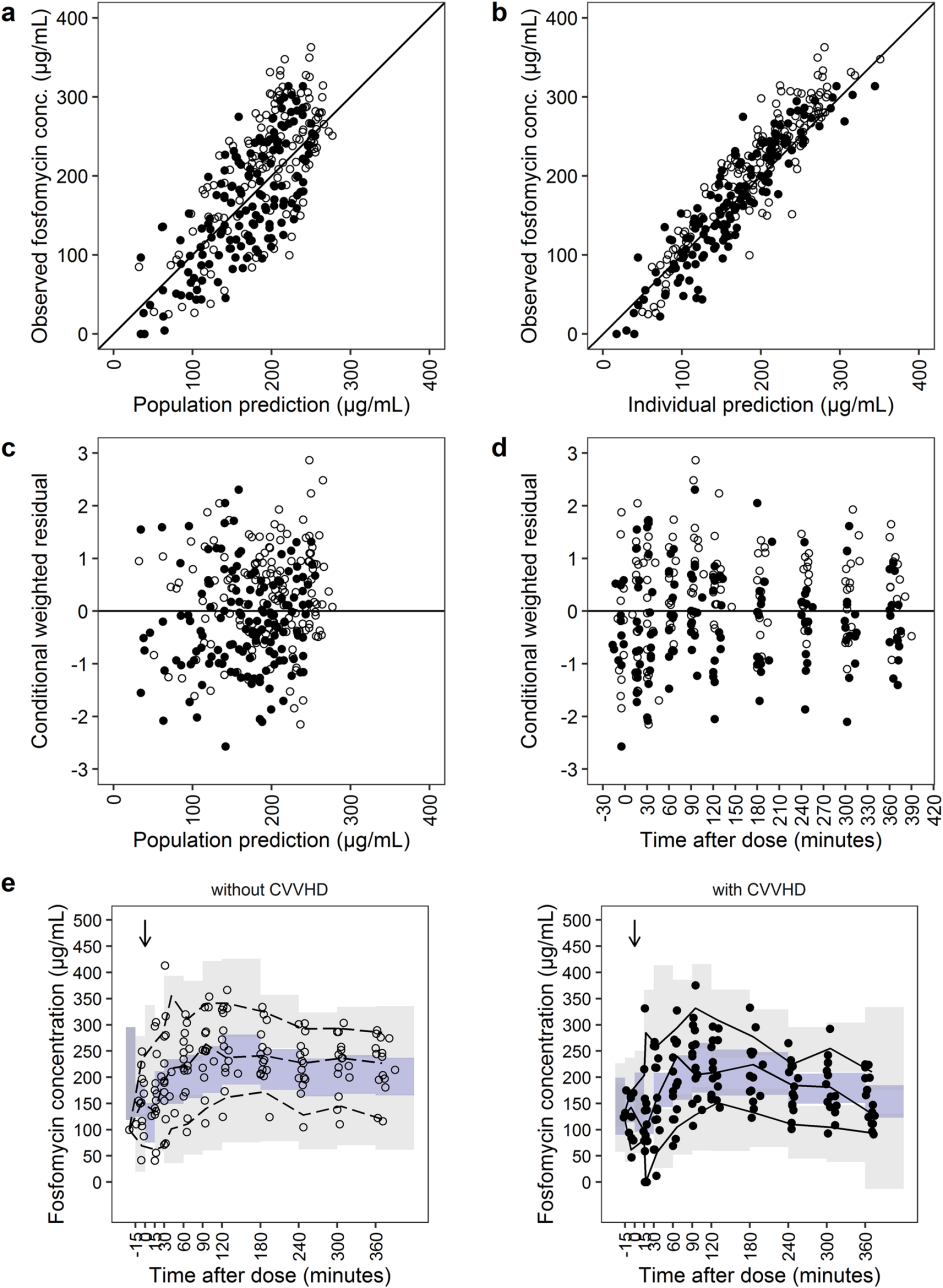
Goodness-of-fit plots and prediction-corrected Visual Predictive Check Goodness-of-fit plots for the final pharmacokinetic model: observed versus population predicted (**a**) and individual predicted (**b**) fosfomycin plasma concentrations. Solid lines indicate lines of identity. Conditional weighted residuals versus population predicted concentrations (**c**) and time after dose (**d**). (**e–f**) Prediction-corrected visual predictive check (pcVPC) for the final pharmacokinetic model without CVVHD (**e**) and with CVVHD (**f**) showing the 95% confidence interval for the predicted median (blue shaded area) and the 95% confidence interval for the predicted 10% and 90% percentiles (grey shaded area). Circles/dots indicate prediction corrected observed plasma concentrations, the dashed/solid lines the median, 10th and 90th percentiles of prediction corrected observed plasma concentrations. Arrows indicate dosing with 5 g of i.v. fosfomycin. (**a–e**) Circles (dashed lines) and dots (solid lines) represent data without and with CVVHD, respectively. conc. = concentration, CVVHD = continuous venovenous hemodialysis.

**Figure 5:**
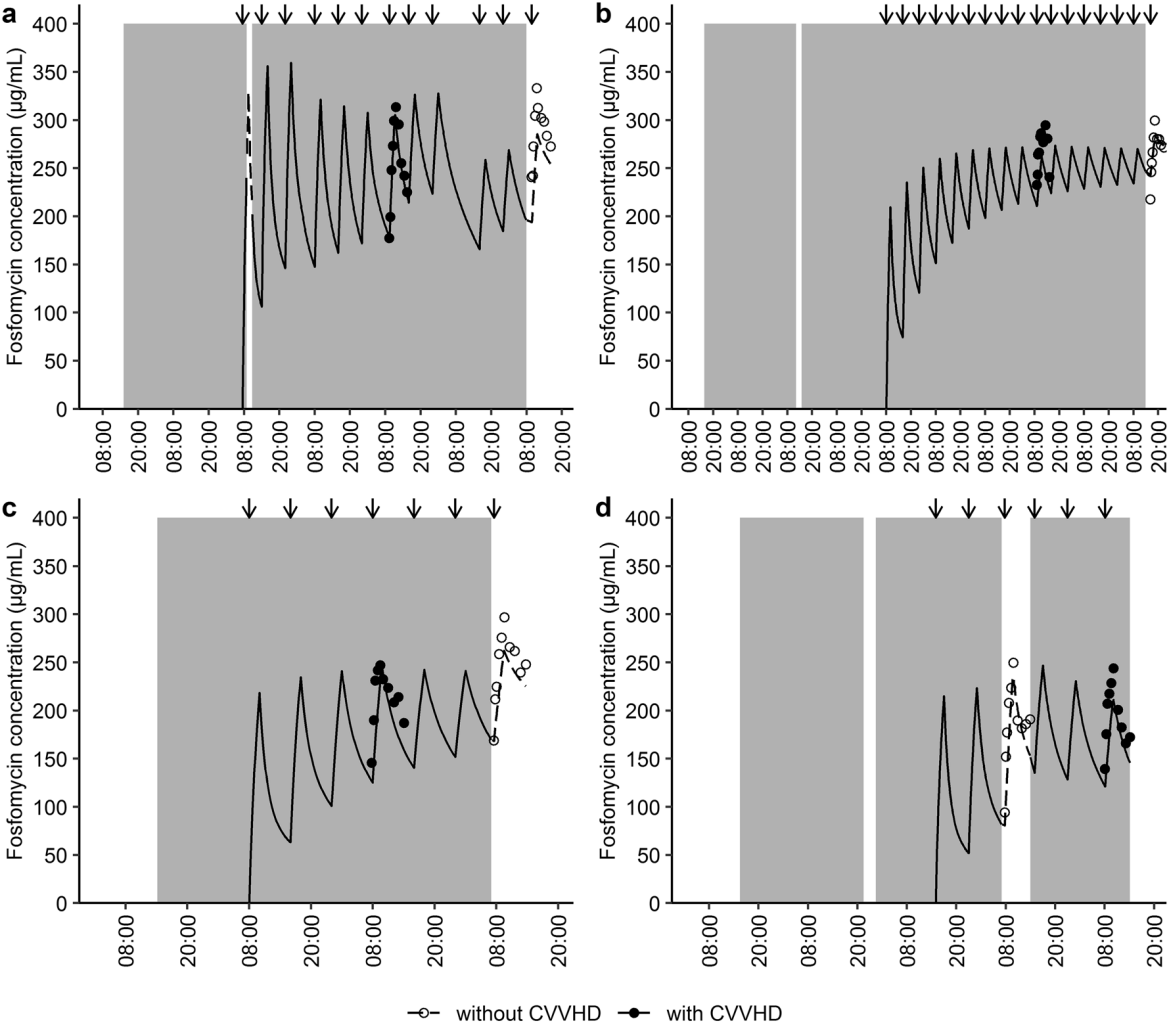
Fosfomycin concentration–time profiles without and with preserved diuresis Exemplary fosfomycin concentration–time profiles of patients without (**a**–**b**) and with (**c**–**d**) preserved diuresis. Circles and dashed lines (white background) represent observed and individually predicted concentrations without CVVHD. Dots and solid lines (grey background) represent observed and individually predicted concentrations during CVVHD. Arrows indicate treatment with 5 g of i.v. fosfomycin. Preserved diuresis (c-d) resulted in considerably lower fosfomycin plasma concentrations. CVVHD = continuous venovenous hemodialysis.

The parameters of the final PK model were estimated with accurate precision (relative standard errors < 30%, Table 2). Goodness-of-fit plots and prediction-corrected visual predictive checks (pcVPC) differentiated by CVVHD treatment are included in Fig. 4. Observed and model predicted fosfomycin concentrations were randomly scattered around the line of identity and conditional weighted residuals showed no trend over time, indicating a good model fit. Exemplary fosfomycin concentration–time profiles of patients with anuria (*n* = 2, Fig. 5a,b) and preserved diuresis (*n* = 2, Fig. 5c,d) demonstrated a good descriptive performance of the PK model. Concentration–time profiles of all patients are presented in Figure S1.

**Table 2:** Parameters of the Final PK Model.

Parameter	Population estimate	RSE (%)
Structural model parameters		
CL_Renal_^a^ [L/h] ($\Theta_{{{\text{CL}}_{{{\text{Renal}}}} }}$)	0.263	16
V_C_^b^ [L] ($\Theta_{{{\text{V}}_{{\text{C}}} }}$)	18.20	23
V_P_ [L] ($\Theta_{{{\text{V}}_{{\text{P}}} }}$)	20.80	27
Q [L/h] ($\Theta_{{\text{Q}}}$)	5.08 ^c^	–
K_0_A^d^ [mL/min] ($\Theta_{{{\text{V}}_{{{\text{K}}0{\text{A}}}} }}$)	0.0288	9
Covariates		
CL_CR_^a^ ($\Theta_{{{\text{CL}}_{{{\text{CR}}}} }}$)	0.0723^c^	–
TSFD ^b^ (Θ_TSFD_)	0.0008	24
Random effects		
IIV V_C_ (%CV)	62.10	16
Additive residual variability [SD; µg/mL]	30.58	16

^a^$${\text{CL}} \; = \; \Theta_{{{\text{CL}}_{{{\text{Renal}}}} }} \; \times \; \left( {1\; + \; \Theta_{{{\text{CL}}_{{{\text{CR}}}} }} \; \times \; {\text{CL}}_{{{\text{CR}}}} } \right) \; + \; {\text{CL}}_{{{\text{CVVHD}}}}$$.

^b^$${\text{V}}_{{\text{C}}} = \Theta_{{{\text{V}}_{{\text{C}}} }} \times (1 + \Theta \;{\text{TSFD}}\; \times \; {\text{TSFD}})\; \times \; {\text{EXP}}\;(\eta_{{{\text{V}}_{{\text{C}}} }} )$$.

^c^Parameters fixed.

^d^Equation 1: ${\text{CL}}_{{\text{CVVHD }}} { = }\frac{{{\text{BFR }}\left( {{\text{e }}^{{\frac{{{\text{K}}_{{0}} {\text{A}}}}{{{\text{BFR}}}}{ }\left( {{\text{1 {-} }}\frac{{{\text{BFR}}}}{{{\text{DFR}}}}} \right)}} { - 1}} \right)}}{{{\text{e }}^{{\frac{{{\text{K}}_{{0}} {\text{A }}}}{{\text{BFR }}}{ }\left( {{\text{1 {-} }}\frac{{{\text{BFR}}}}{{{\text{DFR}}}}} \right){ }}} {\text{{-} }}\frac{{{\text{BFR}}}}{{{\text{DFR}}}}{ }}}{ }$. The following equations describe the fosfomycin concentrations in the central (C1) and peripheral (C2) PK compartments: $\frac{{{\text{dC}}_{1} }}{{{\text{dt}}}} = - \frac{{\text{Q}}}{{{\text{V}}_{{\text{C}}} }} \times {\text{C}}_{1 } + \frac{{\text{Q}}}{{{\text{V}}_{{\text{P}}} }} \times {\text{C}}_{2 } - \frac{{{\text{CL}}}}{{{\text{V}}_{{\text{C}}} }} \times {\text{C}}_{1}$. $\frac{{{\text{dC}}_{2} }}{{{\text{dt}}}} = \frac{{\text{Q}}}{{{\text{V}}_{{\text{C}}} }} \times {\text{C}}_{1} - \frac{{\text{Q}}}{{{\text{V}}_{{\text{P}}} }} \times {\text{C}}_{2}$. CL = total fosfomycin clearance, CLRenal = intrinsic renal fosfomycin clearance, CLCR = urinary creatinine clearance, CLCVVHD = fosfomycin clearance through CVVHD, CV = coefficient of variation, CVVHD = continuous venovenous hemodialysis, IIV = inter-individual variability, K0A = hemodialyzer mass transfer-area coefficient, Q = intercompartmental clearance, RSE = relative standard error, TSFD = time since first dose, VC = central compartment’s volume of distribution at time since first dose of 0 min, VP = peripheral compartment’s volume of distribution, *η* = inter-individual variation.

## Simulations

**Figure 6:**
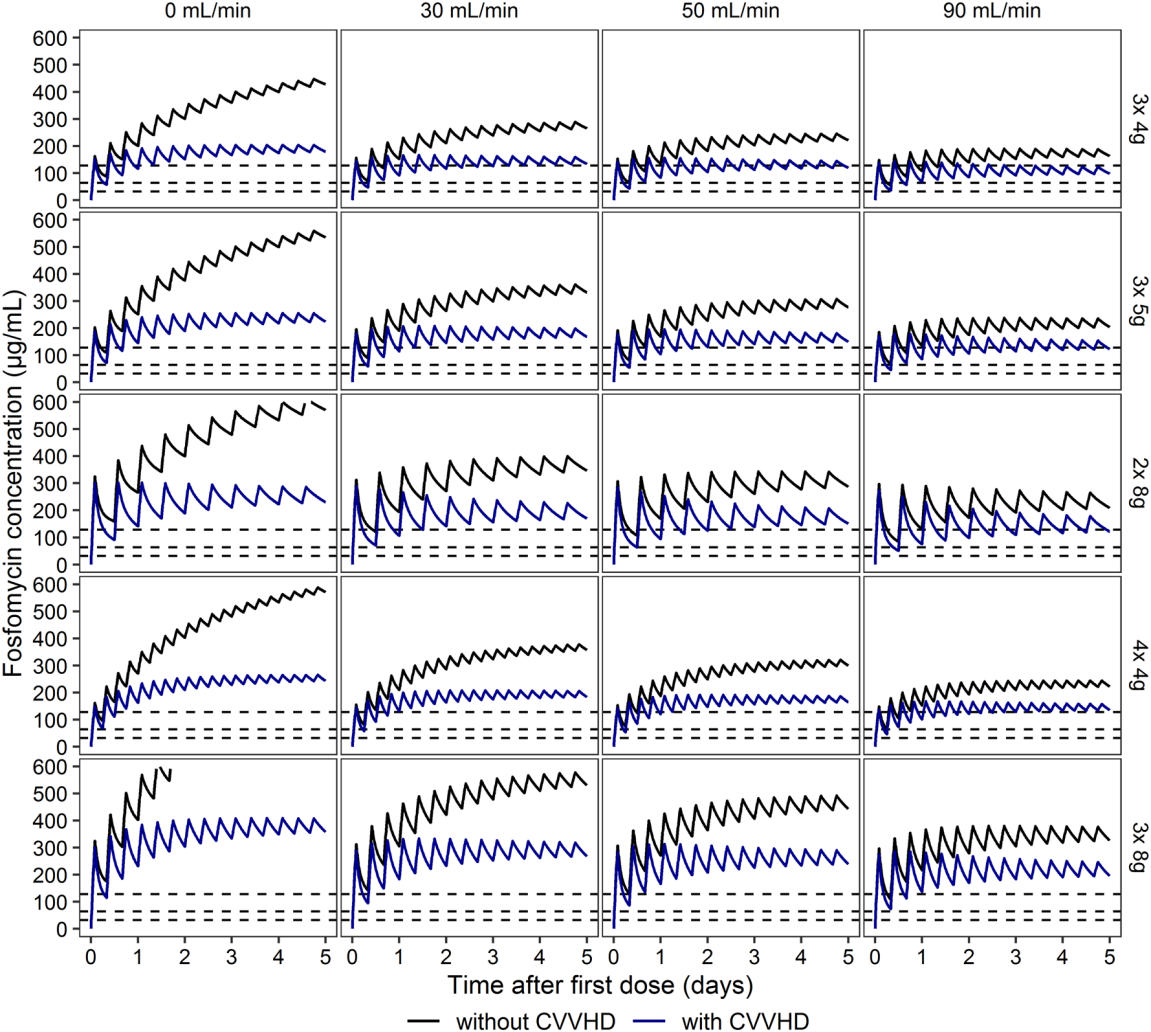
Model-based simulation of fosfomycin plasma concentration Model-based simulation of fosfomycin plasma concentration–time profiles over a period of 5 days after first dose for different dosing regimens (vertical panels) with varying creatinine clearances (horizontal panels) with CVVHD (blue line) and without CVVHD (black line). The dashed lines indicate current EUCAST and CLSI clinical breakpoints or ECOFFs for i.v. fosfomycin of 32, 64 and 128 µg/mL. CVVHD = continuous venovenous hemodialysis.

Model-based simulations of i.v. fosfomycin concentrations are provided in Fig. 6. The scenarios were evaluated using currently valid EUCAST (European Committee on Antimicrobial Susceptibility Testing) and CLSI (Clinical and Laboratory Standards Institute) clinical breakpoints or epidemiological cut-off values (ECOFFs) for i.v. and oral fosfomycin (i.e., 32, 64, and 128 µg/mL). Comparison of CVVHD and no CVVHD treatment in each simulated scenario demonstrate that fosfomycin concentrations were reduced under CVVHD, but adequate concentrations exceeding 64 µg/mL were observed nonetheless (Fig. 6). Furthermore, anuric patients with and without CVVHD displayed no critical accumulation after 5 days. According to our simulations, a minimum dose of 15 g of i.v. fosfomycin across 3 daily doses is required to reach steady state concentrations exceeding 128 µg/mL under CVVHD treatment in patients with a urinary creatinine clearance above 50 mL/min. Furthermore, a minimum dose of 16 g of i.v. fosfomycin across 4 daily doses is required to reach equivalent steady state concentrations in patients with a urinary creatinine clearance above 90 mL/min.

## Discussion

In this observational study of multiple-dose regimes of i.v. fosfomycin in critically ill patients during and following interruption of CVVHD, anuric patients showed hardly any elimination of i.v. fosfomycin without CVVHD. Additionally, concentration–time profiles exhibited considerable interindividual variability. With CVVHD treatment, AUC, c_max_, and t_1/2_ values were reduced by 28.7%, 17.3%, and 51.0%, respectively, resulting in lower i.v. fosfomycin plasma concentrations during renal replacement therapy.

Our results are in accordance with previously published clinical studies. Gattringer et al. determined serum concentrations of i.v. fosfomycin in the arterial and venous line during continuous venovenous haemofiltration (CVVH) after a single 8 g dose in critically ill patients^12^. After 12 h of CVVH, 77% of total i.v. fosfomycin had been removed. Gerecke et al. evaluated plasma concentrations using single-and multiple-dose regimens of i.v. fosfomycin in patients undergoing prolonged intermittent renal replacement therapy (PIRRT) using the GENIUS™ dialysis system^13^. In their study, 74% of the initial dose had been eliminated after 6 h of PIRRT. Dimski et al. determined i.v. fosfomycin concentrations in critically ill patients with acute kidney injury during intermittent hemodialysis (iHD; GENIUS™ dialysis system) on a treatment regimen of 5 g thrice daily^14^. iHD treatment immediately after the first dose resulted in serum fosfomycin concentrations below 32 mg/L, the current EUCAST breakpoint for i.v. fosfomycin in *Enterobacterales* and *Staphylococcus* spp infections. Nevertheless, iHD treatment following subsequent doses of i.v. fosfomycin resulted in acceptable concentrations above 32 mg/L.

Based on our clinical data, we developed a thorough population pharmacokinetic model for patients with and without CVVHD treatment. Using the Michaels equation, our data were most accurately described by a two-compartment model with renal and dialysis clearance, the latter of which represents an additional pathway for non-renal elimination of fosfomycin. Nevertheless, fosfomycin is excreted over the feces in healthy subjects. It is possible that this elimination pathway becomes more important in renal failure. In the absence of data discriminating the amount of fosfomycin which is eliminated by either renal elimination, non-renal elimination, or feces, however, we assumed that fosfomycin elimination results from renal and non-renal clearance.

Our model is comparable to another published model by Parker et al.^9^. They developed a PK model for i.v. fosfomycin based on clinical data from critically ill patients with multiple dose regimens, but without renal replacement therapy. In their study, patients’ remaining renal function was better than in our cohort, which is reflected in a greater mean creatinine clearance (59.0 mL/min [calculated using the Cockcroft-Gault formula] vs. 20.7 mL/min [measured from 12 h urine output]). This could explain the difference in estimated renal clearance of fosfomycin, which was considerably higher in their model (2.06 L/h on the first day vs. 0.26 L/h). Weight is an important variable of the Cockcroft-Gault formula, and patients included by Parker et al. were substantially less heavy than our cohort (mean weight 71.5 kg vs. 90.0 kg). Consequently, a group comparison based on calculated creatinine clearance, which would relatively overestimate patients’ remaining renal function in our cohort, would be biased and unreliable. Both studies found similar population parameter estimates for the volumes of the central compartment (27.2 L vs. 18.2 L) and the peripheral compartment (22.3 L vs. 20.8 L), whereas intercompartmental clearance was estimated to be slightly higher (19.8 L/h vs. 5.08 L/h) than in our study. Unsurprisingly, both analyses concur that renal clearance of fosfomycin was influenced by patients’ remaining renal function. In our analysis, urinary creatinine clearance accounted for a substantial amount of fosfomycin elimination. The relative contribution of dialysis clearance to total fosfomycin elimination was dependent upon patients’ creatinine clearance. A creatinine clearance exceeding 120 mL/min resulted in a small contribution of dialysis to total fosfomycin elimination, while dialysis clearance accounted for the majority of total fosfomycin elimination once creatinine clearance fell below 30 mL/min. This may be relevant in patients whose indication for hemodialysis is not anuria (e.g., hypervolemia, metabolic acidosis), or in patients whose renal function recovers during treatment with i.v. fosfomycin. In these cases, underdosing could result in inadequate plasma levels below EUCAST clinical breakpoints for relevant pathogens. However, convection techniques such as hemofiltration and hemodiafiltration are more appropriate in patients whose renal replacement therapy indication is hypervolemia. Furthermore, our analysis suggests that central compartment volume increases with time following administration of the initial i.v. fosfomycin dose, which could be explained by an increase in extracellular fluid with disease progression in critical ill patients.

Based on previously published PK studies, fosfomycin dosage recommendations can be extrapolated for specific patient populations depending on the level of remaining renal function. For example, Gattringer et al. concluded that 8 g of fosfomycin every 12 h might be an appropriate dose for patients undergoing CVVH^12^. Gerecke et al. recommended 5 g of i.v. fosfomycin thrice daily, replenished at the end of the dialysis session^13^. Dimski et al. recommended an induction dose of 8 g of i.v. fosfomycin, followed by a maintenance dose of 5 g of i.v. fosfomycin in anuric patients with iHD^14^.

Our final population PK model was utilized for simulations. Using our model, we determined that approved daily doses of 12–24 g of i.v. fosfomycin are unlikely to result in critical accumulation or underdosing within 5 days of the initial dose in anuric patients with or without CVVHD treatment. In patients undergoing CVVHD treatment with a urinary creatinine clearance above 50 mL/min, i.v. fosfomycin dosage should be increased to at least 15 g across three daily doses. Similarly, in patients with a urinary creatinine clearance above 90 mL/min, a minimum of 16 g of fosfomycin should be administered across four daily doses. Using these recommendations, sufficient plasma concentrations of fosfomycin (> 64 mg/L) will likely be achieved in patients with and without CVVHD, independently of residual renal function.

Our study has several limitations. First, we determined plasma concentrations of fosfomycin. Thus, we cannot comment on the penetration of infected tissue at different doses. Microdialysis of tissue biopsies could have helped clarify this question. Second, the small number of patients may have prevented further covariates from being identified for our final PK model. Consequently, covariates should be re-analyzed in a larger cohort. Third, we determined the total fosfomycin concentration in patient plasma. Although binding to albumin is generally low^15^, including plasma protein binding in the analysis may have influenced the results. Fourth, we did not measure fosfomycin concentrations in the dialysate or urine. Additional information, such as the concentration of fosfomycin in the dialysate, could possibly have improved our model. However, such data are not usually collected in routine clinical practice. More importantly, even though the observed data are highly variable, our chosen model describes the observed plasma concentrations of fosfomycin very well. Finally, taking daily weights to quantify edema might have influenced the modeling as well. However, in this study, the dosage of i.v. fosfomycin was not weight-adjusted.

## Conclusions

The PKs of i.v. fosfomycin in critically ill patients with need for CVVHD show great variability. Preserved diuresis results in considerably lower plasma concentrations of fosfomycin. A two-compartment population pharmacokinetic model comprising renal and dialysis clearance adequately describes i.v. fosfomycin concentrations in critically ill patients with CVVHD. Urinary creatinine clearance accounts for a substantial amount of fosfomycin elimination. Model-based simulations suggest a safe and effective use of 12–24 g of i.v. fosfomycin across two or three daily doses for five days in anuric patients with or without CVVHD. In patients with CVVHD and a creatinine clearance above 50 (90) mL/min, doses should be increased to at least 15 (16) grams across 3 (4) daily doses.

## Methods

### Ethics

This prospective observational PK study was registered with German Clinical Trials (ID DRKS00017450) and was approved by the regional ethics committee (Ärztekammer Saarland, Saarbrücken, Germany, Identification Number 117/17). The study was conducted in accordance with relevant guidelines and regulations. Written informed consent was obtained from patients or their legal representatives.

### Subject selection

This single-center study included critically ill patients suffering from infection with i.v. fosfomycin-susceptible bacteria and from renal insufficiency. They were at least 18 years old, and they had a body mass index of less than 35 kg/m^2^ and a central venous line for other medical reasons. Exclusion criteria were lack of informed consent, pregnancy or breastfeeding.

### Protocol and sample collection

Patients received 5 g of fosfomycin (InfectoFos®, Infectopharm, Heppenheim, Germany) intravenously over 120 min every 8 h. Blood samples were drawn from a central venous line 5 min before and subsequently 15, 30, 60, 90, 180, 240, 300 and 360 min after the start of the infusion. Samples were taken from each patient both with and without CVVHD, the latter referring to the interruption of treatment required for re-installation of a new tubing system.

Creatinine, albumin, total protein, urea, potassium and sodium were determined daily in serum. In patients with preserved diuresis, urinary creatinine clearance was calculated daily from urine collected over the course of 12 h (Eq. 2):

2 $$ Urinary \,Creatinine\, Clearance \left[ {\frac{ml}{{\min }}} \right] = \frac{{Urine\, Creatinine \left[ {\frac{mg}{{dl}}} \right]*Urine\, Volume \left( {ml} \right)}}{{Plasma \,Creatinine \left[ {\frac{mg}{{dl}}} \right]*Collection\, Time \left[ {\min } \right] }} $$

### CVVHD

Patients were treated with CVVHD (multiFiltrate Ci-Ca, Fresenius Medical Care, Bad Homburg, Germany) using polysulfone membrane hemofilters (Ultraflux AV 1000S; Fresenius Medical Care). The dialysate solution was free of calcium and phosphate (Ci-Ca Dialysate K4, Fresenius Medical Care) and had a potassium concentration of 4 mmol/L. Regional citrate anticoagulation (Sodium citrate 4%; Fresenius Medical Care) was used with calcium re-substitution (Calcium chloride 100 mL, Fresenius Medical Care) to compensate calcium losses. BFR was 100 mL/min and DFR was 2 L/h initially. Subsequently, BFR and DFR were adjusted individually to optimize fluid status, electrolytes and diuresis.

### Measurements

The chromatographic separation was performed on an Agilent 1260 series HPLC chromatograph (Santa Clara, USA) with a 3.0 × 150 mm XBridge BEH Amide XP 2.5 μm column protected by a 2.1 × 5 mm XBridge BEH Amide XP 2.5 μm VanGuard Cartridge at 35 °C and a flow rate of 0.35 mL/min. The mobile phase contained 4 mM ammonium formate in (A) water pH = 5 and (B) acetonitrile 90% at pH = 5. The linear gradient had the following program: 0 min (0:100) → 4 min (45:55) → 9 min (45:55) → 10 min (0:100) → 16 min (0:100). A 6130B quadrupole mass spectrometer (Agilent) was used for the detection and quantification of all compounds. All data of i.v. fosfomycin were acquired in negative single ion mode (SIM) by electrospray ionization at m/z =  − 137. The spray chamber was set to the following parameters: fragmentor at 75, capillary voltage at − 3 kV, drying gas flow at 12 L/min, nebulizer pressure at 35 psi, and drying gas temperature at 350 °C.

For sample preparation, a 10 μL sample was mixed with 10 μL of water, 20 μL of buffer solution (2% ammonium formate pH = 5) and 360 μL of mobile phase B (pH = 5), and was then centrifuged at 4000 g for 10 min at 4 °C. Subsequently, the supernatant was used for analysis. The HPLC injection volume was 40 μL.

The calibration was conducted with a 10 mg/mL i.v. fosfomycin (InfectoPharm, Heppenheim, Germany) standard solution prepared in water and diluted to provide calibration standards of 25, 50, 100, 150, 200, 300, 400, 500, 600 and 700 μg/mL. All calibration standards were diluted in drug-free plasma. Calibration curves were calculated by plotting the concentration of the calibration standard against the measured peak area. The peak areas were evaluated using OpenLab CDS C.01.05 (Agilent). All curves yielded a correlation coefficient of at least R^2^ > 0.985. All back‐calculated concentrations of the standard samples displayed a maximum deviation of ± 15% from the nominal value.

### Software

Statistical analyses were performed, and graphics were created using R (version 3.6.3, R Foundation for Statistical Computing, Vienna, Austria) and R Studio (version 1.4.1717, RStudio, Inc., Boston, MA, USA). Pharmacokinetic modeling and simulation were performed using a non-linear mixed-effects modeling technique implemented in the software NONMEM® (Version 7.4, ICON Development Solutions, Ellicott City, MD, USA). Data are presented as means ± SD.

### Non-compartmental analysis

NCA was performed using the PKNCA R package (version 0.9.4). The area under the concentration–time curve (AUC) was calculated from the first to the last measurement within a series of PK measurements utilizing the PKNCA::pk.calc.auc function. Elimination half-life (t_1/2_) was computed using the PKNCA::pk.calc.half.life function, with the measurement at the time of maximum concentration (c_max_) allowed to be included and a minimum of three measurements to be included for calculation. Results were compared to CVVHD treatment. Group comparisons were graphically evaluated using ladder plots, and statistically evaluated using a two-sided paired t-Test or Wilcoxon signed-rank test, when indicated, each with a significance level of *p* < 0.05.

### Population pharmacokinetic modeling

First-order conditional estimation with interaction was used. Model selection was based on the NONMEM® objective function (− 2 log likelihood, Objective Function Value OFV), graphical evaluation by means of goodness-of-fit plots, and precision of parameter estimates. A reduction of 3.84 points (*p* < 0.05) in OFV indicated a statistically significant difference between nested models. The same criteria were applied for the inclusion of covariates. The final model was evaluated via pcVPC based on 1000 replicates of the original dataset^16^.

One-, two- and three-compartment models were fitted initially. An additional non-renal clearance (CL_CVVHD_) was added to determine the amount of fosfomycin eliminated under CVVHD conditions. In patients without preserved diuresis, intrinsic fosfomycin elimination was fixed to zero. Preserved diuresis was defined as a residual diuresis exceeding 100 mL in 24 h. Interindividual variability was implemented using exponential random effect models. Several statistical models were tested to describe residual variability.

Time after first dose, age, weight, creatinine clearance and serum levels of creatinine, albumin, total protein, urea, potassium, and sodium were tested as covariates to determine their influence on interindividual variability of the estimated model parameters.

### Simulations

Dosing scenarios within the approved daily doses of 12–24 g of i.v. fosfomycin were simulated for 5 days based on the final PK model: 4, 5 and 8 g thrice daily, 8 g twice daily and 4 g four times daily. For each dosing regimen, urinary creatinine clearances of 0, 30, 50 and 90 mL/min were simulated to reflect various grades of renal insufficiency. BFR was set to 100 mL/min and DFR was set to 2 L/h. To provide individual dosing recommendations, the simulated fosfomycin concentrations were evaluated against the PK/PD target 100% Time > ECOFF (128 µg/mL).

### Supplementary Information

Supplementary Information.

## Data Availability

The datasets used and analyzed during the current study are available from the corresponding author on reasonable request.
