# Supplementary material for: Population pharmacokinetic modeling of multiple-dose intravenous fosfomycin in critically ill patients during continuous venovenous hemodialysis
Source: Sci Rep. 2023 Oct 24;13:18132. doi: 10.1038/s41598-023-45084-5 (PMC10598009; doi:10.1038/s41598-023-45084-5)
Supplement: Supplementary file 1 — Supplementary Information. [file 41598_2023_45084_MOESM1_ESM.docx]

**Supplement**

**Figure S1**


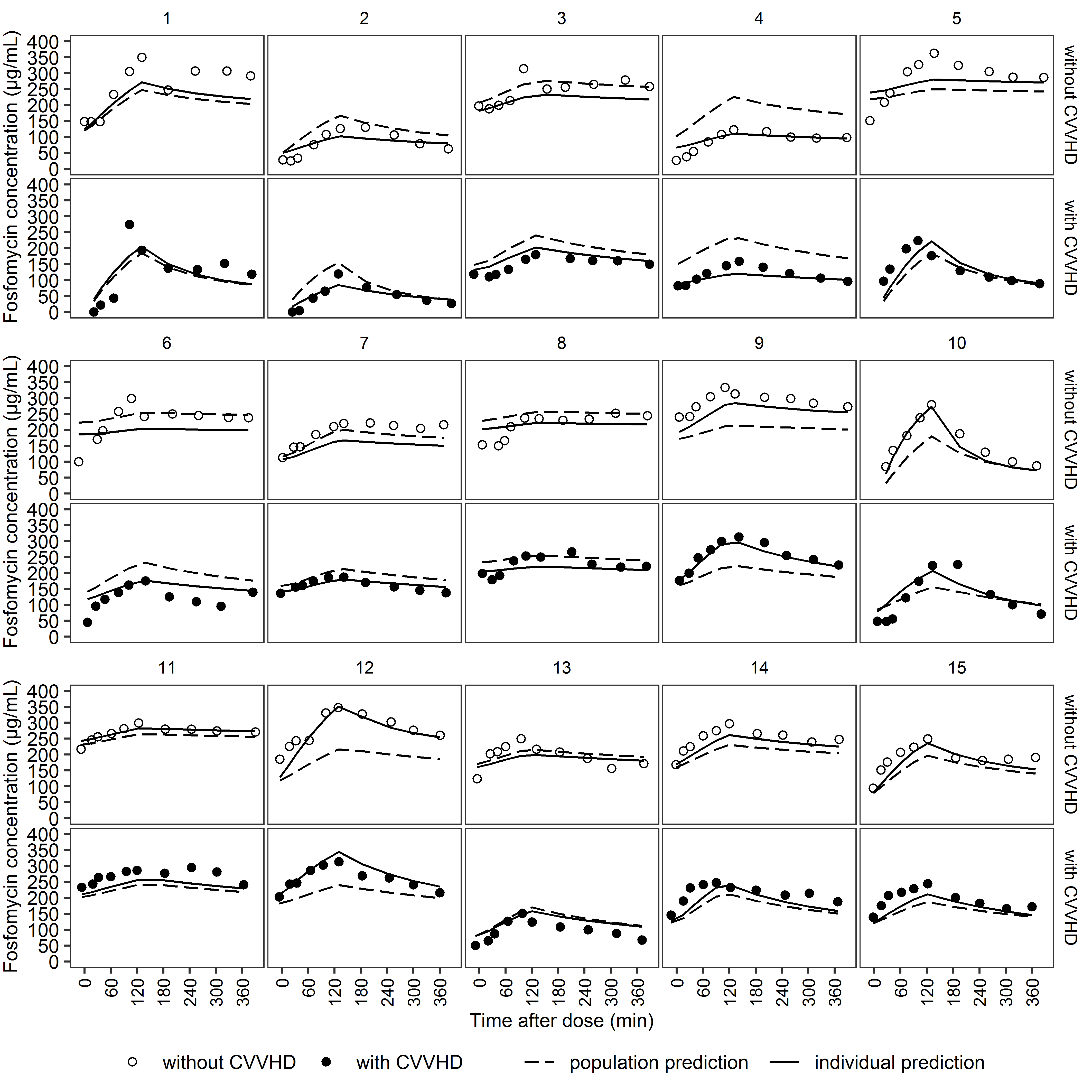


**Figure S1** Goodness-of-fit plots showing individual fosfomycin plasma concentrations-time profiles of 15 patients. Circles and dots represent observed concentrations without and with CVVHD, respectively. Dashed and solid lines represent population and individual predicted concentrations, respectively. CVVHD = continuous venovenous hemodialysis
